# The prognostic value of RV strain in patients with heart failure with reduced ejection fraction on sacubitril/valsartan: a critical appraisal and future directions

**DOI:** 10.1093/eschf/xvaf016

**Published:** 2026-01-19

**Authors:** Jian Wu, Yihua He

**Affiliations:** Echocardiography Medical Center, Beijing Anzhen Hospital, Capital Medical University, No. 2 Anzhen Road, Chaoyang District, Beijing 100029, China; Echocardiography Medical Center, Beijing Anzhen Hospital, Capital Medical University, No. 2 Anzhen Road, Chaoyang District, Beijing 100029, China

We commend Pastore *et al*.^1^ for their insightful sub-analysis of the DISCOVER-ARNI registry, which highlights the prognostic value of right ventricular free wall longitudinal strain (fwRVLS) in patients with heart failure with reduced ejection fraction (HFrEF) treated with sacubitril/valsartan. The findings underscore the potential of advanced echocardiographic parameters in refining risk stratification and personalizing treatment in this population. However, we would like to raise several methodological and conceptual points that may help contextualize the results and suggest directions for future research.

## 1. Study design and sample size limitations

The retrospective nature of this sub-analysis, despite prospective follow-up, inherently introduces the risk of selection bias. The reduction of the original cohort from 341 to 136 patients (a 60% decrease) due to missing speckle-tracking echocardiography (STE) data represents a significant limitation. The exclusion of patients based on acoustic window quality may have inadvertently selected for a population with better image quality—potentially those with less advanced disease or favourable body habitus—thus limiting the generalizability of the results to the broader HFrEF population. Moreover, the non-significant difference in ΔfwRVLS between groups (*P* = .06) may reflect inadequate power due to sample size reduction rather than a true absence of effect. To strengthen the validity of such findings, future studies should adopt a prospective design with pre-specified STE acquisition protocols to maximize data completeness. Additionally, employing statistical methods such as multiple imputation—assuming data are missing at random—could mitigate selection bias and enhance the reliability of the results.^2^

## 2. Population homogeneity and generalizability

The study population was exclusively Italian and predominantly male (82%). While this reflects certain epidemiological characteristics of HFrEF, it may limit the external validity of the proposed fwRVLS cut-off value of −20% across diverse ethnicities, healthcare systems, and sex subgroups.^3^ The underrepresentation of women remains a recurring issue in cardiovascular trials and may obscure sex-specific differences in disease progression or treatment response. To improve generalizability, international multicentre collaborations would be valuable in validating these findings across varied populations. Furthermore, incorporating interaction analyses by sex within multivariate models could help clarify whether the prognostic utility of fwRVLS remains consistent between men and women.

## 3. Mechanistic depth and multimodal validation

Although the study establishes a compelling association between fwRVLS and clinical outcomes, the underlying pathophysiological mechanisms connecting right ventricular dysfunction to adverse events remain insufficiently explored. Right ventricular performance is modulated by a complex interplay of factors, including afterload conditions (such as pulmonary artery systolic pressure, sPAP), ventricular interdependence, and left ventricular filling pressures.^4,5^ Therefore, future investigations would benefit greatly from incorporating multimodal imaging protocols—for instance, combining echocardiography with CMR in a subgroup of patients—to cross-validate STE-based fwRVLS and elucidate its relationship with myocardial fibrosis, loading conditions, and contractile reserve. Furthermore, given that the right ventricular wall is thinner and more sensitive to loading variations than the left, integrating load-adjusted indices such as right ventricular-pulmonary arterial coupling (e.g. TAPSE/sPAP ratio) could provide a more physiologically nuanced assessment than strain alone.^6^

Particularly relevant in this context is the potential role of right ventricular myocardial work, which integrates deformation (right ventricular global longitudinal strain, RV GLS) with afterload (sPAP).^7,8^ This novel parameter may offer a more comprehensive and load-independent measure of RV mechanical efficiency. Its application seems especially promising in patients receiving sacubitril/valsartan, given the drug’s established effects in reducing secondary pulmonary hypertension through improved cardiac function. Assessing myocardial work could potentially uncover treatment-related improvements in right ventricular performance that are not captured by RV GLS alone, thereby offering superior prognostic insight and a more refined tool for monitoring therapeutic response.

In conclusion, the study by Pastore *et al*. represents a valuable contribution to the field by identifying fwRVLS as a promising prognostic marker in HFrEF patients undergoing ARNI therapy. However, the retrospective design, sample size limitations, and lack of mechanistic and multimodal validation suggest that these results should be interpreted as hypothesis-generating. We encourage the authors and the broader research community to build upon these findings through prospective, diverse, and mechanistically oriented studies to further validate and integrate advanced echocardiographic parameters into clinical decision-making.

## Data Availability

There are no new data generated.
